# Supplementation of diet with non-digestible oligosaccharides alters the intestinal microbiota, but not arthritis development, in IL-1 receptor antagonist deficient mice

**DOI:** 10.1371/journal.pone.0219366

**Published:** 2019-07-08

**Authors:** Rebecca Rogier, Thomas H. A. Ederveen, Harm Wopereis, Anita Hartog, Jos Boekhorst, Sacha A. F. T. van Hijum, Jan Knol, Johan Garssen, Birgitte Walgreen, Monique M. Helsen, Peter M. van der Kraan, Peter L. E. M. van Lent, Fons A. J. van de Loo, Shahla Abdollahi-Roodsaz, Marije I. Koenders

**Affiliations:** 1 Experimental Rheumatology, Radboudumc, Nijmegen, The Netherlands; 2 Centre for Molecular and Biomolecular Informatics, Radboudumc, Nijmegen, The Netherlands; 3 Danone Nutricia Research, Utrecht, The Netherlands; 4 Laboratory of Microbiology, Wageningen University, Wageningen, The Netherlands; 5 NIZO food research, Ede, The Netherlands; 6 Division of Pharmacology, Utrecht institute for Pharmaceutical Sciences, Utrecht University, Utrecht, The Netherlands; University of Illinois, UNITED STATES

## Abstract

The intestinal microbiome is perturbed in patients with new-onset and chronic autoimmune inflammatory arthritis. Recent studies in mouse models suggest that development and progression of autoimmune arthritis is highly affected by the intestinal microbiome. This makes modulation of the intestinal microbiota an interesting novel approach to suppress inflammatory arthritis. Prebiotics, defined as non-digestible carbohydrates that selectively stimulate the growth and activity of beneficial microorganisms, provide a relatively non-invasive approach to modulate the intestinal microbiota. The aim of this study was to assess the therapeutic potential of dietary supplementation with a prebiotic mixture of 90% short-chain galacto-oligosaccharides and 10% long-chain fructo-oligosaccharides (scGOS/lcFOS) in experimental arthritis in mice. We here show that dietary supplementation with scGOS/lcFOS has a pronounced effect on the composition of the fecal microbiota. Interestingly, the genera *Enterococcus* and *Clostridium* were markedly decreased by scGOS/lcFOS dietary supplementation. In contrast, the family Lachnospiraceae and the genus *Lactobacillus*, both associated with healthy microbiota, increased in mice receiving scGOS/lcFOS diet. However, the scGOS/lcFOS induced alterations of the intestinal microbiota did not induce significant effects on the intestinal and systemic T helper cell subsets and were not sufficient to reproducibly suppress arthritis in mice. As expected, we did observe a significant increase in the bone mineral density in mice upon dietary supplementation with scGOS/lcFOS for 8 weeks. Altogether, this study suggests that dietary scGOS/lcFOS supplementation is able to promote presumably healthy gut microbiota and improve bone mineral density, but not inflammation, in arthritis-prone mice.

## Introduction

Rheumatoid arthritis (RA) is a systemic autoimmune disease characterized by chronic joint inflammation and progressive destruction of cartilage and bone. Inflammatory cells such as T cells, B cells and macrophages accumulate in the inflamed joint, which results in synovitis and tissue destruction [1]. Although the exact etiology is unknown, RA is considered to be driven by genetic as well as environmental factors [1]. Several recent studies have shown that the composition of intestinal microbiota is perturbed in patients with new-onset as well as chronic RA [2–5]. This suggests that the microbiome may be an environmental factor that can influence the development of RA.

RA patients have increased levels of T helper-17 (Th17) cells in their peripheral blood mononuclear cells [6]. These Th17 cells are considered to be a major pathogenic mediator in RA, as these cells produce IL-17, a potent inducer of matrix metalloproteinases and proinflammatory cytokines such as interleukin-(IL)6 and IL-8. [6–9]. In addition, regulatory T (Treg) cells, which normally downregulate inflammation, were shown to have decreased suppressive activity in RA patients [10]. Intestinal microbiota strongly influences immune homeostasis and by altering the Th17/Treg cell balance the development of autoimmune diseases in mice [11–14]. Several studies have shown that development and severity of spontaneous arthritis in K/BxN and IL-1 receptor antagonist deficient (IL-1Ra^-/-^) mice is strongly reduced in germ-free (GF) mice [11, 15, 16]. In addition, colonizing arthritis prone SKG mice with *Prevotella*-dominated microbiota of RA patients resulted in increased intestinal Th17 levels and aggravated arthritis development compared with mice receiving microbiota of healthy controls [17]. Furthermore, colonizing mice with the human gut commensal *Prevotella histicola* suppressed Th17 responses and the development of inflammatory arthritis after immunization with collagen type II [14]. These observations suggest that the intestinal microbiota plays an important role in the development of autoimmune arthritis, which makes modulation of the intestinal microbiota an interesting novel approach to suppress autoimmunity.

Prebiotics, defined as non-digestible carbohydrates that selectively stimulate the growth and activity of beneficial microorganisms, provide a relatively non-invasive approach to modulate the intestinal microbiota [18]. Dietary supplementation with a prebiotic mixture of 90% short-chain galaco-oligosaccharides and 10% long-chain fructo-oligosaccharides (scGOS/lcFOS) is known to particular promote the growth of beneficial bacteria such as bifidobacteria and lactobacilli [19–21]. In addition, several animal and clinical studies demonstrated that dietary supplementation with scGOS/lcFOS suppresses acute allergic symptoms, a process dependent on the induction of Treg cells [22–26]. Furthermore, multiple studies showed a beneficial effect of scGOS/lcFOS on bone mineral density [27–31]. Something which could be beneficial in the context of RA, as bone mineral density has been shown to be reduced in RA patients [32, 33].

The aim of the current study was to assess the efficacy of microbiota modulation using scGOS/lcFOS as a therapeutic approach for T cell-dependent autoimmune experimental arthritis in IL-1Ra^-/-^ mice, which develop spontaneous arthritis due to excessive IL-1 receptor signaling [34]. We recently reported that IL-1Ra deficiency results in reduced diversity and richness, and causes specific taxonomic alterations characterized by increased *Helicobacter* spp. and decreased *Ruminococcus* spp. and *Prevotella* ssp., which specifically induces Th17 differentiation in intestinal lamina propria [16]. In addition, tobramycin-induced alterations of commensal intestinal microbiota suppressed arthritis in IL-1Ra^-/-^ mice [16].

In this study we describe a significant increase in the bone mineral density after mice were on a diet supplemented with 5% scGOS/lcFOS for 8 weeks. Using high-throughput 16S rRNA marker gene sequencing, we here show that dietary supplementation with scGOS/lcFOS had a pronounced effect on the composition of the fecal microbiota. However, scGOS/lcFOS-induced alterations of the intestinal microbiota did not induce any significant beneficial effects on the intestinal and systemic T helper cell subsets and were unable to reproducibly suppress arthritis.

## Materials and methods

### Mice

IL-1Ra deficient mice on BALB/c background were kindly provided by Dr. M. Nicklin (Sheffield, England). The mice were housed in filter-top cages under specific pathogen-free conditions and the water and food were provided *ad libitum*. Age- and gender-matched littermates were used in all experiments, the average age at the start of the experiments was 8 weeks. Development of arthritis was scored macroscopically by two blinded observers using an arbitrary scoring system as follows; 0, no redness and swelling; 0.25, slight redness; 0.5, slight redness and swelling; 0.75–1, mild redness and swelling; 1.25–1.5, moderate redness and swelling; 1.75–2, severe redness and swelling. Only hind paws were scored, because arthritis development in the front paws is rare in this model [35]. Littermates reaching the inclusion score of 0.5–1.0 of arthritis were split, regrouped with animals of the same sex, and randomly divided over the different treatment cages with different scGOS/lcFOS-containing food pellets provided. All animal procedures were approved by the ethics committee of the Radboud University Medical Center and were performed according to the appropriate codes of practice (approval number RU-DEC2010-082).

### Prebiotic diet

The groups either received standard AIN-93 synthetic feed control diet or a diet supplemented with a mixture of scGOS (Vivinal GOS, Borculo Domo, Zwolle, The Netherlands) and lcFOS (Raftiline HP, Orafti, Wijchen, The Netherlands) at a ratio of 9:1. The experimental diets contained either 1%, 2.5% or 5% scGOS/lcFOS added to standard AIN-93 synthetic feed (Research Diet Services, Wijk bij Duurstede, The Netherlands). The mice stayed on their respective diets for 8–10 weeks.

### Microbiota sequencing and data analysis

After 8 weeks of dietary intervention, feces were collected and fecal bacterial DNA was isolated using phenol/chloroform-based extraction method combined with bead-beating [36]. As described in detail previously [16], sequencing was performed by DNAVision (Charleroi, Belgium) on a Roche 454 GS-FLX System using 16S rRNA bar-coded primers targeting the V5-V6 conserved DNA regions (forward primer 784F: 5’-AGGATTAGATACCCTGGTA-3’, reverse primer 1061R: 5’-CRRCACGAGCTGACGAC-3’) [37]. For gene sequence analysis, a customized workflow based on Quantitative Insights Into Microbial Ecology (QIIME version 1.2) was adopted (http://qiime.org/) [38]. Settings recommended in QIIME 1.2 tutorial were applied. Additionally, reads were filtered for chimeric sequences using Chimera Slayer as described before [39]. Operational taxonomic unit (OTU) clustering was performed with settings as recommended by QIIME [40] using an identity threshold of 97%. The Ribosomal Database Project classifier version 2.2 was used for taxonomic classification [41]. Hierarchical clustering of samples was performed using the average distances between samples with weighted UniFrac as distance measure as implemented in QIIME. For statistical analysis and generation of figures, QIIME implemented R-packages, SciPy [42] (www.Scipy.org), Graphpad Prism version 5.0, and Microsoft Office Excel 2007 were adopted.

#### Histology

For histological assessment of arthritis, total ankle joints were isolated and fixed in 4% formaldehyde for 4 days, thereafter decalcified in 5% formic acid and embedded in paraffin. Tissue sections of 7μm were stained using Haematoxylin & Eosin to study synovial inflammation, chondrocyte death and cartilage and bone erosion. Safranin O staining was performed on the sections to determine proteoglycan depletion. Each parameter was scored on a scale from 0–3 in a blinded manner.

### Lymphocyte isolation

Mice were sacrificed by cervical dislocation, immediately followed by isolation of the popliteal lymph nodes (pLN) and small intestine (SI). pLNs were disrupted on a 70 μm cell strainer, and the cells were collected in RPMI-1640 (Gibco; Invitrogen) supplemented with 10% FCS and gentamycin (50mg/l, Centrafarm). The SI was placed in ice-cold PBS and mesenteric fat and Peyer’s patches were removed. This was followed by incubation with 33 mM EDTA on ice for 30 minutes to remove epithelial cells, and subsequent digestion with 1 mg/ml collagenase-D (Roche) and 10 μg/ml DNAse I (Sigma) at 37°C for three cycles of 15 minutes. Lamina propria lymphocytes (LPLs) were then harvested at the interphase of a 40:80% Percoll gradient (Sigma), washed thoroughly and stimulated and stained as described below.

### Flow cytometry

LPLs and pLN cells stimulated for 4 hours with PMA (50 ng/ml; Sigma), ionomycine (1 μg/ml; Sigma), and the Golgi-traffic inhibitor Brefeldin (1 μl/ml; BD Biosciences). Cells were stained with anti-CD3-PE (BD Pharmingen) or anti-CD3-APC (eBioscience) and anti-CD4-APC (Biolegend) or anti-CD4-FITC (BD Pharmingen). Next, the cells were fixed and permeabilized using fixation/permeabilization buffer (eBioscience). For intracellular staining the cells were incubated in permeabilization buffer (eBioscience) containing anti-IL-17-FITC (Biolegend), anti-IFNγ-FITC (BD Pharmingen), anti-IL-4-PE (BD Pharmingen) or Foxp3-FITC (eBioscience). An appropriate isotype matched control antibody was used in all FACS analyses. Cells were analyzed on a FACS Calibur using the CellQuest software (BD Biosciences). Results were analyzed with FlowJo version 7.6.5.

### RNA isolation and quantitative real-time polymerase chain reaction (qPCR)

Tissues were homogenized using a MagNA Lyser instrument (Roche). RNA was isolated in TRIzol reagent (Sigma) as described before [15]. Quantitative real-time PCR (qRT-PCR) was performed using the StepOne System (Applied Biosystems) using the SYBR green Master Mix (Applied Biosystems). Primer sequences were as follows: for GAPDH (House-keeping gene), 5′-GGCAAATTCAACGGCACA-3′ (forward) and 5′-GTTAGTGGGGTCTCGCTCTG-3′ (reverse); for T-bet 5’-CAACAACCCCTTTGCCAAAG-3’ (forward) and 5’-TCCCCCAAGCAGTTGACAGT-3’ (reverse); for RORγt 5’-CTGTCCTGGGCTACCCTACTGA-3’ (forward) and 5’-AAGGGATCACTTCAATTTGTGTTCTC-3’ (reverse); for FoxP3 5’-AGGAGAAGCTGGGAGCTATGC-3’ (forward) and 5’-GGTGGCTACGATTGCAGCAA-3’ (reverse); for IFNγ 5’-TCTTCTTGGATATCTGGAGGAACTG-3’ (forward) and 5’-AGAGATAATCTGGCTCTGCAGGAT-3’ (reverse); for IL17a 5’-CAGGACGCGCAAACATGA -3’ (forward) and 5’- GCAACAGCATCAGAGACACAGAT -3’ (reverse); for IL10 5’-ATTTGAATTCCCTGGGTGAGAA-3’ (forward) and 5’-ACACCTTGGTCTTGGAGCTTATTAA-3’ (reverse).

### Dual-energy X-ray absorptiometry (DEXA) scanning

To assess the effect of scGOS/lcFOS on bone mineral density, dual-energy X-ray absorptiometry (DEXA, Lunar PIXImus) scanning was performed after 10 weeks of treatment. A whole-body scanner and specifically designed software for small animals was used as described previously [43]. The mice were anesthetized for the duration of the procedure by exposure to 2.5% isoflurane-oxygen gas via a nose cone. One scan per mouse was performed and bone mineral density (g/cm^2^) was calculated with PIXImus software. The head was excluded from the calculations using a manual region of interest.

#### Statistics

Differences in the relative abundance of bacterial taxa between treatment groups were evaluated using Mann-Whitney U test. We corrected for multiple testing using the Benjamini and Hochberg procedure with false discovery rate (FDR) set at 25%, and differences with a *p-*value < 0.05 which passed the FDR test were considered statistically significant. Kruskal-Wallis with a Dunn’s post-test was used to compare cell levels, arthritis histology scores, gene-expression and bone mineral density between treatment groups. For arthritis scores, two-tailed Mann-Whitney U test was performed for area under the curve.

## Results

### Prebiotic diet containing scGOS/lcFOS alters the composition of intestinal microbiota in IL-1Ra^-/-^ mice

To determine the effect of a prebiotic diet containing scGOS/lcFOS on the intestinal microbiota, IL-1Ra^-/-^ mice were fed either a control diet, or a diet containing 1 or 2.5% scGOS/lcFOS for 8 weeks. The diet was well tolerated and did not cause any growth retardation or weight loss. 16S rRNA marker gene pyrosequencing was performed on DNA from fecal samples collected after 8 weeks of intervention to identify changes in the intestinal microbiota. The average sequencing depth, total number of reads and operational taxonomic units (OTU) were not affected by the scGOS/lcFOS diet and remained comparable between the experimental groups (S1 Table).

**Fig 1:**
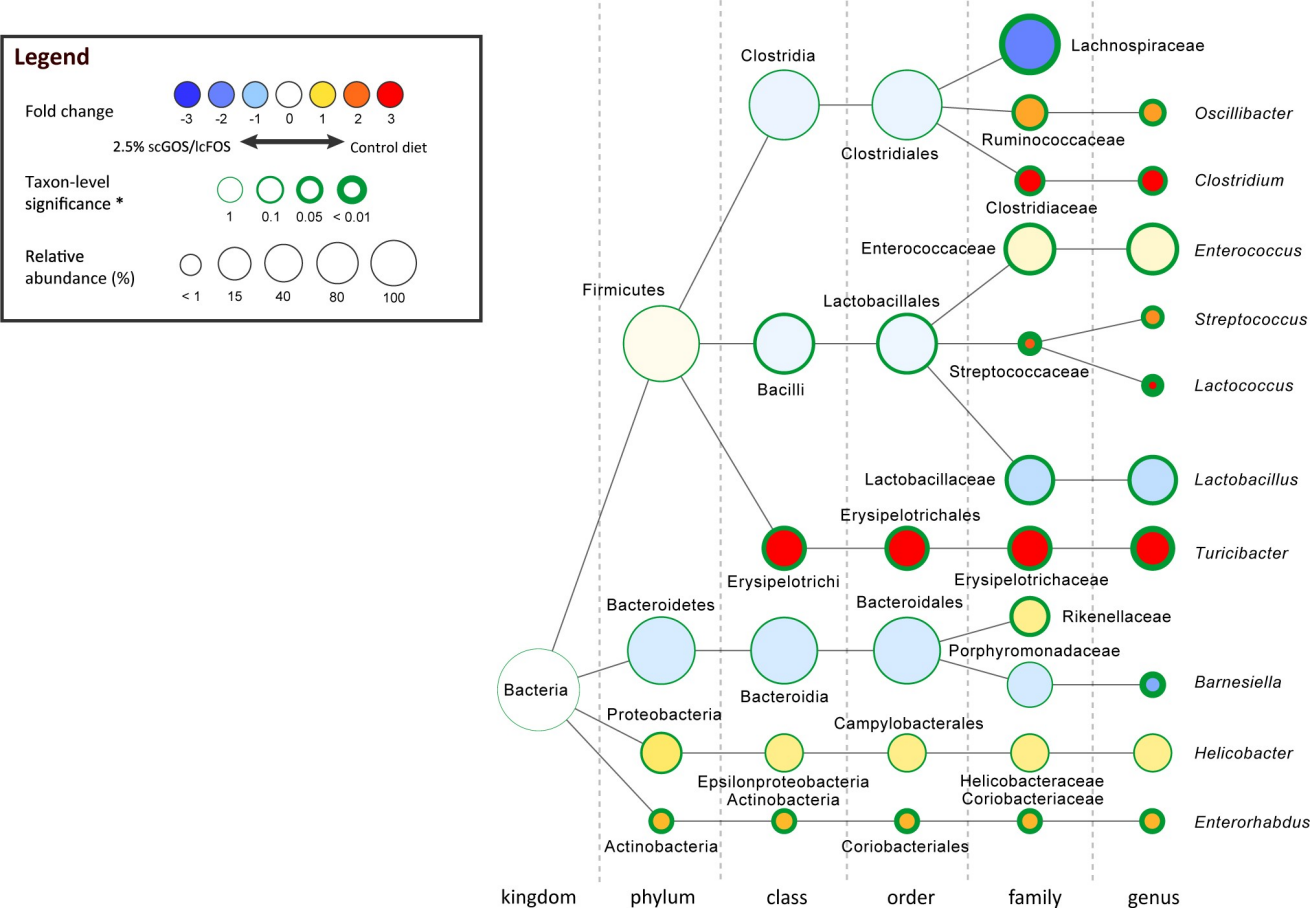
Prebiotic diet containing scGOS/lcFOS significantly alters the composition of intestinal microbiota of IL-1Ra^-/-^ mice. Phylogenetic tree created by Cytoscape software showing specific changes in intestinal microbial community at different taxonomic levels in the mice fed 2.5% scGOS/lcFOS diet compared to mice fed a control diet. Nodes represent taxa, and the size of each node represents its relative abundance. The color blue indicates an increase in the 2.5% scGOS/lcFOS fed mice compared to control mice, while the color red indicates an decrease in the 2.5% scGOS/lcFOS fed mice. The thickness of the green border indicates the degree of statistical significance by Mann-Whitney U test, uncorrected.

Furthermore, we did not observe any significant changes in the number of observed species, Chao1 index, Shannon index or phylogenetic distance whole tree metric (S1A–S1C Fig). In addition, principal coordinates analysis (PCoA) based on weighted UniFrac distances showed no clear differences between the different groups (S1D Fig). Although we did not observe any significant effect on bacterial richness and diversity, the scGOS/lcFOS diet significantly altered the composition of the intestinal microbiota. A prominent effect observed in the 2.5% scGOS/lcFOS fed mice compared to the control group was a highly significant increase in the family Lachnospiraceae (Fig 1 and S2 Table). However, the resolution of the 16S gene pyrosequencing was not sufficient to identify the genera within the family Lachnospiraceae that were increased in the 2.5% scGOS/lcFOS fed mice (Fig 1 and S2 Table).

A significant increase in the genus *Lactobacillus* was observed for mice receiving the 2.5% scGOS/lcFOS, corroborating results observed previously by Vos *et al*. (Fig 1 and S2 Table) [20]. The genus *Barnesiella* (family Porphyromonadaceae) was increased as well in the 2.5% scGOS/lcFOS group (Fig 1 and S2 Table), although still represented a low abundant taxon. A significant near complete elimination of bacteria belonging to the genus *Turicibacter* (family Erysipelotrichaceae) was observed in the 2.5% scGOS/lcFOS fed mice (Fig 1 and S2 Table). In addition, the genera *Oscillibacter* (family Ruminococcacea), *Enterococcus* (family Enterococcaceae), *Streptococcus* (family Streptococcaceae), *Lactococcus (*family Streptococcaceae) and *Clostridium (*family *Clostridiaceae)* were significantly decreased in the 2.5% scGOS/lcFOS group (Fig 1 and S2 Table), although none of these taxa were highly dominant among the microbiota. None of the observed differential abundant taxa in the 2.5% scGOS/lcFOS group were found to be significant in the group receiving the 1% scGOS/lcFOS diet; however, the fold changes for the 1% scGOS/lcFOS group correlated significantly with the change for the 2.5% scGOS/lcFOS group (Spearman rank test: rho 0.45, p-value 0.003). Altogether, these data show that a 2.5% scGOS/lcFOS diet alters the composition of the intestinal microbiota.

### Treatment of arthritic IL-1Ra^-/-^ mice with scGOS/lcFOS diet has no effect on the progression of experimental arthritis

**Fig 2:**
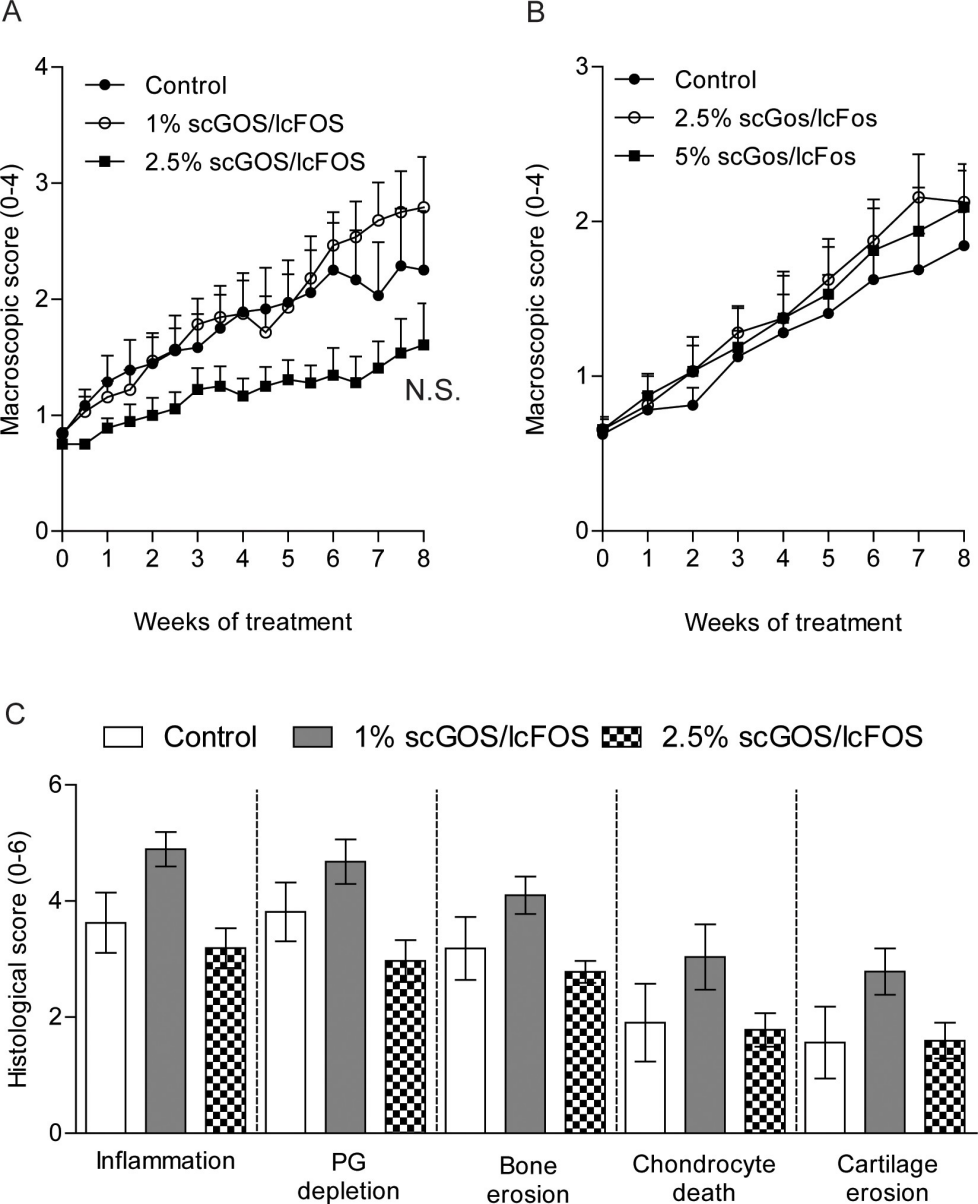
Oral treatment of arthritic IL-1Ra^-/-^ mice with prebiotic scGOS/lcFOS has no effect on the progression of arthritis. (A-B) Arthritis severity scores (0–2 per paw) of IL-1Ra^-/-^ mice fed a control diet or a diet containing either 1% or 2.5% scGOS/lcFOS (A) or 2,5 or 5% scGOF/lcFOS (B) for 8 weeks. (C) Histological scores of synovial inflammation, proteoglycan (PG) depeletion, bone erosion, chondrocyte (chond.) death and cartilage erosion. Data shown mean + SEM of 8–9 mice per group. Treatment started when mice had a score of 0.75–1. NS = not-significant (*p* = 0.0571) as tested by Kruskal-Wallis with Dunn’s post test.

To determine the efficacy of scGOS/lcFOS in the treatment of joint inflammation as well as cartilage and bone destruction during experimental arthritis, IL-1Ra^-/-^ mice with ongoing arthritis under conventional microbial status were orally fed a control diet or a diet containing 1% or 2.5% scGOS/lcFOS for 8 weeks. The severity of arthritis over time was comparable between the group receiving the 1% scGOS/lcFOS diet and the control group. The mice in the 2.5% scGOS/lcFOS group showed a trend toward reduced arthritis severity scores over the entire 8-week study period; however, this effect was not significant (*p* = 0.0571; Fig 2A). Aiming to maximize the observed effects of the dietary scGOS/lcFOS supplement, we replicated the experiment with IL-1Ra^-/-^ mice receiving either 2.5% or 5% scGOS/lcFOS supplemented diets. However, this study showed no effect of the prebiotic diet on arthritis scores at any dose (Fig 2B). Histological examination of the ankle joints confirmed this lack of therapeutic efficacy of the scGOS/lcFOS diet and revealed no significant effects on inflammation, bone and cartilage damage (Fig 2C).

**Fig 3:**
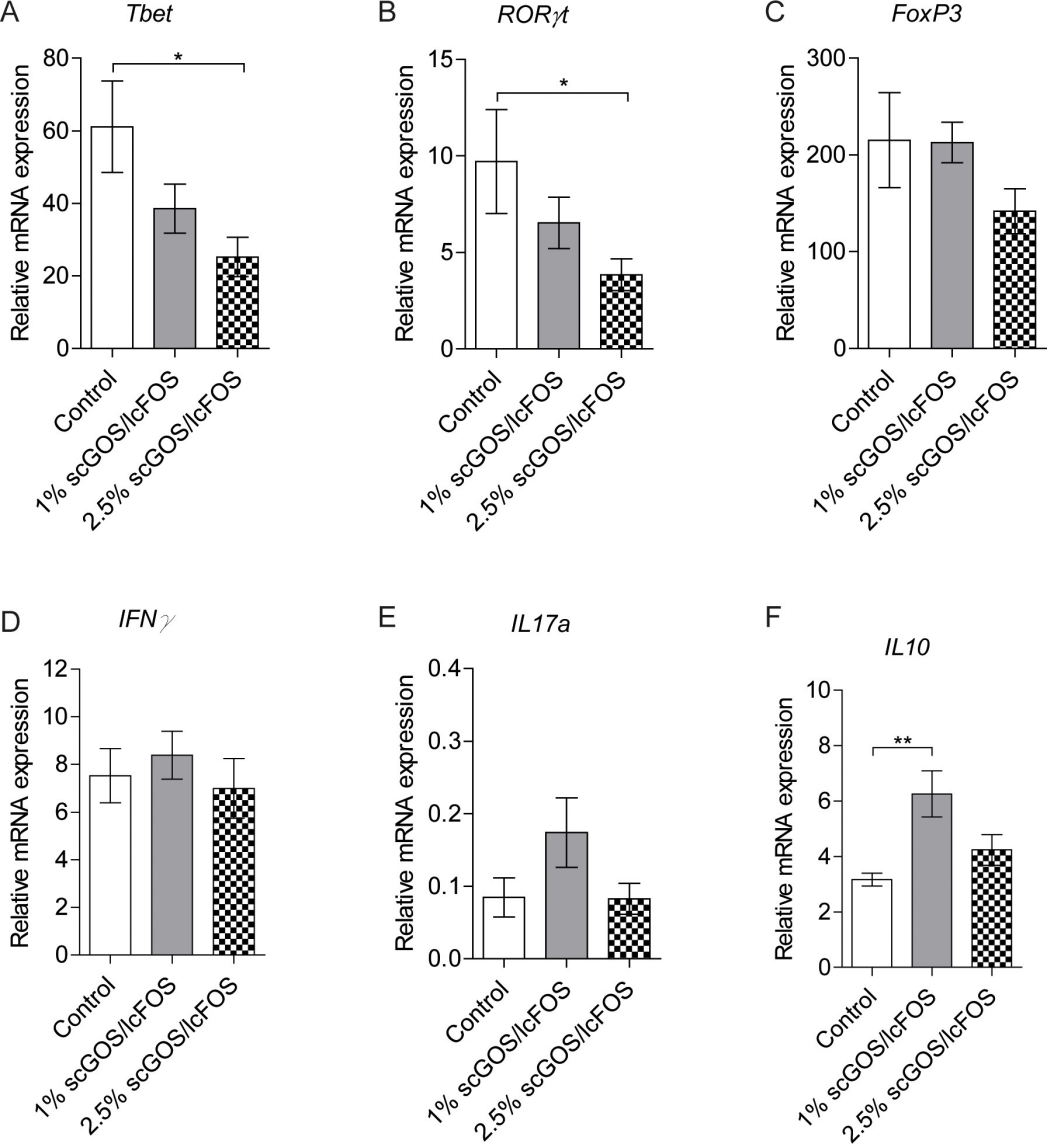
Oral treatment of arthritic IL-1Ra^-/-^ mice with prebiotic scGOS/lcFOS has no effect on T cell subsets during arthritis. (A-B) Gene expression of Tbet (A), ROR*γ*t (B) and FoxP3 (C) in joint draining lymph nodes of arthritic IL-1Ra^-/-^ mice fed a control diet (n = 5) or a diet containing either 1% (n = 5) or 2.5% (n = 8) scGOS/lcFOS. Relative mRNA expression is shown as 2^-ΔCt^ *10000, corrected for GAPDH. *p<0.05 by Kruskal-Wallis with Dunn’s post test.

To determine the effect of the different scGOS/lcFOS diets on the local T cell response, we determined the gene expression of the transcription factors *Tbet*, *RORγt* and *FoxP3* and cytokines *IFNγ*, *IL17a* and *IL10* (relevant for Th1, Th17 and Tregs, respectively) in pLNs, which drain the arthritic ankle joint. The gene expression of *Tbet* and *RORγt* was significantly reduced in pLNs of the mice which received the 2.5% scGOS/lcFOS diet compared to the control mice (Fig 3A, 3B, 3D and 3E). However, this was not reflected and supported by a reduction in the expression of *IFNγ* and *IL17a*. Furthermore, the expression of Treg-related *FoxP3* was also not affected by the scGOS/lcFOS diet, whereas IL-10 expression was only increased in the 1% scGOS/lcFOS diet group (Fig 3C and 3F).

In addition, we isolated cells from the draining lymph nodes and performed flow cytometric analysis. This analysis showed no effect of the different scGOS/lcFOS doses on the abundance of Th1, Th2, Th17 and Treg cells in pLNs (S2A–S2D Fig). Based on these data, we conclude that scGOS/lcFOS-induced alterations of the intestinal microbiota were not sufficient to significantly alter the joint-associated T helper cells subsets and reproducibly suppress arthritis.

### Prebiotic diet containing 5% scGOS/lcFOS diet significantly improves bone mineral density

Because of lack of clear therapeutic effects in the first experiment with 1% and 2.5% scGOS/lcFOS, we included a secondary readout parameter as positive control in the study with 2.5% and 5% scGOS/lcFOS. For this, bone mineral density was added as additional readout. It has previously been described that scGOS/lcFOS diet can increase intestinal mineral absorption from diet and thereby improve bone mineral density in rats [27–31]. Therefore, we performed DEXA scanning to measure bone mineral density in our mice. This revealed that a prebiotic diet containing 5% scGOS/lcFOS significantly improves the overall bone mineral density of IL-1Ra^-/-^ mice (S4A Fig). The bone mineral content (BMC) also tended to be increased in the 5% scGOS/lcFOS treated mice; however, this increase was statistically not significant (S4B Fig). This finding indicates that the scGOS/lcFOS diet has a beneficial effect on bone mineral density during experimental arthritis, and that despite the lack of anti-arthritic effects, the scGOS/lcFOS levels were sufficient to have systemic effects in these mice.

### scGOS/lcFOS diet has no effect on intestinal T helper cell subsets in IL-1Ra^-/-^ mice

Intestinal microbiota are known to greatly influence the balance between pro-inflammatory and regulatory mucosal T cell responses [44]. Considering the observed effects of scGOS/lcFOS on the intestinal microbiota, we investigated the gene expression of the transcription factors *Tbet*, *RORγt* and *FoxP3* relevant for differentiation of Th1, Th17 and Tregs, respectively, in ileum, mesenteric lymph nodes (mLN) and spleen of IL-1Ra^-/-^ mice fed 1% and 2.5% scGOS/lcFOS diet. We observed no effect of the scGOS/lcFOS diet on expression levels of these genes in any of the tissues we tested (S4A–S4C Fig). However, *FoxP3* mRNA expression in the colon of 2.5% and 5% scGOS/lcFOS fed mice was slightly, but not significantly, increased compared to mice on a control diet (S4D Fig).

In addition, we analyzed the effect of the 2.5% and 5% scGOS/lcFOS diet on T helper cell subset in the small intestine lamina propria with flow cytometry. The small intestine lamina propria (SI-LP) of mice on the 5% scGOS/lcFOS diet contained slightly increased percentages of Th17, Th1 and Tregs, however these effect failed to reach statistical significance (S5A–S5C Fig). In contrast, the percentage of IL-4 producing Th2 cells present in the SI-LP showed a non-significant reduction in the 5% scGOS/lcFOS group compared to the control group (S5D Fig). We conclude from these data that although a scGOS/lcFOS diet significantly affected the intestinal microbiome, it did not alter mucosal T helper cell subsets in intestinal lamina propria.

## Discussion

Recent developments in the fields of microbiome research and immunology have shown that intestinal microbiota play a critical role in the maintenance of immune homeostasis [45–47]. Therefore, modulation of the intestinal microbiota may offer an interesting novel approach to suppress autoimmunity. In this study, we assessed the efficacy of microbiota modulation using a specific prebiotic mixture as a therapeutic approach in experimental arthritis.

For the study presented here we used IL-1Ra^-/-^ mice which spontaneously develop arthritis due to excessive IL-1 receptor signaling [34]. We have previously shown that arthritis development in these mice is highly dependent on the intestinal microbiome as arthritis is strongly attenuated under germ-free conditions [15, 16]. In the current study we show that a 2.5% scGOS/lcFOS dietary supplementation had no significant effects on the microbial richness or diversity in IL-1Ra^-/-^ mice; however, it resulted in an altered composition of the intestinal microbiota. This was most notably characterized by a significant increase in *Lachnospiraceae* spp. and *Lactobacillus* ssp.. Members of the family *Lachnospiraceae* have recently been linked to alleviation of experimental encephalomyelitis [48]. It was hypothesized that the increase in *Lachnospiraceae* resulted in an increased production of intestinal butyrate [48]. Butyrate is a short chain fatty acid known to induce differentiation of Treg cells and reduce colonic inflammation [49–52]. In addition, a recent study showed that the composition of microbiota prior to arthritis onset differs between the collagen induced arthritis (CIA)-susceptible and CIA–resistant mice [53]. This study found that *Lachnospiraceae* was more abundant in CIA-resistant mice, while *Lactobacillaceae* was more abundant in CIA-susceptible mice [53]. Furthermore, *Lachnospiraceae* was found to be decreased in gut microbiota of psoriatic arthritis patients [54]. In our study, however, the increase in *Lachnospiraceae* did not result in a significant suppression of IL-1Ra^-/-^ arthritis.

In addition, it has been reported that *Clostridium difficile*-infected mice with a microbiota dominated by *Lachnospiraceae* developed a milder disease [55]. Another clinical study showed that the presence of *Lachnospiraceae* was associated with lower risk of *Clostridium difficile* infection in adult recipients of allogeneic hematopoietic stem cells transplantation [56]. Furthermore, imbalances observed in the gut microbiota of inflammatory bowel disease patients was characterized by reduced abundance of *Lachnospiraceae* [57]. These studies suggest a beneficial role for *Lachnospiraceae* in gut health and protection against pathogens.

In mice, scGOS/lcFOS dietary supplementation also resulted in an increased prevalence of fecal bifidobacteria and lactobacilli [20]. In accordance with these studies, we observed an increase of 2.83% in *Lactobacillus* in the 2.5% scGOS/lcFOS fed mice in comparison to control diet, however bifidobacteria were absent in our IL-1Ra^-/-^ mice and could therefore not be affected in our study. Added to infant formulas, scGOS/lcFOS has been described to stimulated the growth of bifidobacteria and lactobacilli and reduce the numbers of pathogenic bacteria [19, 58, 59]. In addition, a recent paper described that infants receiving scGOS/lcFOS supplemented formula showed increased *Bifidobacterium* and decreased *Clostridium* and *Lachnospiraceae* [60]. In agreement with this study *Clostridium* was decreased in the scGOS/lcFOS treated mice in our studies; however, we observed a strong increase in the family *Lachnospiraceae*. Therefore, the effects observed in our study differ markedly from the effects observed in infants, suggesting that the effect of scGOS/lcFOS depends on the host and endogenous microbiome at start of treatment.

In this study we show that bone mineral density is increased in mice fed a diet supplemented with 5% scGOS/lcFOS. This is in agreement with previous studies which showed that a scGOS/lcFOS mixture increases mineral absorption and bone mineral density in rats [27–31]. Similar to our current study, these studies observed an increase in the abundance of *Lactobacillus* in the rats receiving the scGOS/lcFOS supplemented diet [28]. In addition, those studies reported a reduction in cecal pH values [27, 28]. It was therefore hypothesized that the scGOS/lcFOS diet increased the production of organic acids (short chain fatty acids and lactic acids) by lactic acid bacteria such as *Lactobacillus*, which lowers the pH and thereby improves mineral absorption [27]. However, since we do not have 16S data of mice receiving 5% scGOS/lcFOS we do not know which bacteria are responsible for this effect in our study. Altogether, the significant improvement of the bone mineral density suggests that the scGOS/lcFOS prebiotic mixture has beneficial effects on the bone in the context of arthritis.

We previously showed that the aberrant microbiota in IL-1Ra^-/-^ mice specifically induced IL-17 production by intestinal lamina propria lymphocytes, an effect that could be transferred to wild-type mice by fecal microbiota [16]. Previous studies showed that a scGOS/lcFOS containing diet enhanced the percentage of Th1 cells and tended to reduce Th2 response in mice [61, 62]. In another study it was shown that suppression of the allergic responses by scGOS/lcFOS depends on the presence of CD25+ Tregs [22, 23]. Furthermore, lactobacilli are thought to induce Treg differentiation by modulating dendritic cell function [63]. In addition, butyrate produced by Lachnospiraceae could also induce Treg differentiation [49]. This suggests that a scGOS/lcFOS diet and subsequent increase in *Lactobacillus* and *Lachnospiraceae* could cause an anti-inflammatory shift in Th cell responses. However, analysis of the intestinal lamina propria lymphocytes with flow cytometry in our study did not show any effect on Th cell subsets. This suggests that despite the effect of scGOS/lcFOS on the intestinal microbiota, the diet did not result in modulation of the intestinal immune response in IL-1Ra^-/-^ mice. Excessive IL-1 signaling is known to downregulate TGF-β-induced Foxp3 expression and enhance Th17 differentiation [64]. The lack of modulation of Th cells and arthritis development in our studies could be due to the enhanced IL-1 signaling in IL-1Ra deficient mice, overruling the immune suppressive effects of the scGOS/lcFOS-modulated microbiota.

Although we did not find convincing evidence for an improvement of host immune (proinflammatory) responses by effect of scGOS/lcFOS prebiotics, the observed strong lactobacillogenic effect is in line with literature [19–21]. Interestingly, others have reported certain Lactobacillus species to be associated with RA, which raises the question whether a depletion of these taxa could potentially ameliorate arthritis onset or progression. For example, Zang et al. described a dysbiosis in the microbiota of gut and oral niches from RA patients, based on shotgun metagenomics sequencing data, and specifically reports an overrepresentation of *L*. *salivarius* at these sites [3, 65]. Liu et al. has also found significantly more *Lactobacillus* in the fecal microbiota of RA compared to healthy controls [66]. They furthermore showed in a CIA mouse model that oral pretreatment with strains of *L*. *salivarius* and *L*. *plantarum* isolated from RA patients was able to reduce the arthritis phenotype in a Th17-dependent manner [67]. Intriguingly, in that same study they reported a reduction in bone erosion in CIA mice treated with the lactobacilli. In conclusion, although lactobacilli are generally considered beneficial gut commensals for the host, it can be assumed that different *Lactobacillus* species or strains exert different (immune) responses in the context of RA. Unfortunately, technical limitations in short-length 16S rRNA marker-gene sequencing does not allow for confidently classifying microbiota to the level of (sub)species. Therefore, we cannot speculate on the different subsets of lactobacilli that were present in our samples.

Another possibility is that the specific microbiota modulated by the scGOS/lcFOS diet were not relevant to the ongoing inflammatory processes and that the Th17-driving bacteria were not affected. We recently demonstrated that IL-1Ra deficiency reduces the intestinal microbial diversity and richness, and causes specific alterations in composition of the intestinal microbiota [16]. The taxonomic alterations in IL-1Ra^-/-^ mice were characterized by overrepresentation of the genera *Helicobacter*, *Rikenella*, *Butyricimonas* and *Streptococcus*, while the genera *Prevotella*, *Parasutterella*, *Xylanibacter*, *Ruminococcus*, *and Barnesiella* were underrepresented in the IL-1Ra^-/-^ mice compared to the WT mice [16]. Interestingly, in the 2.5% scGOS/lcFOS fed mice *Streptococcus* were decreased and *Barnesiella* was increased compared to the control group. This might suggest that a 2.5% scGOS/lcFOS diet can partly restore the dysregulated microbiota of IL-1Ra^-/-^ mice. Treatment of IL-1Ra^-/-^ mice with tobramycin significantly reduced arthritis severity and resulted in a near-complete elimination of *Helicobacter* and a highly significant reduction of *Clostridium* [16]. In this current study, 2.5% scGOS/lcFOS diet did not have a strong effect on *Helicobacter*, as only a small non-significant decrease was observed in the 2.5% scGOS/lcFOS treated group (3.13% in control group vs. 2.08% in 2.5% scGOS/lcFOS group). However, scGOS/lcFOS treatment did significantly reduce *Clostridium* abundance (Fig 1), which was also one of the genera significantly affected by tobramycin treatment. This suggests that bacteria which contribute to the progression of arthritis in IL-1Ra^-/-^ mice are only partly affected by scGOS/lcFOS supplementation.

## Conclusions

Prebiotics such as scGOS/lcFOS have potential benefits in providing nutrient sources to specific beneficial bacteria to promote a diverse and healthy gut microbiota. In our study, we observed an increase in *Lactobacillus* genus and Lachnospiraceae family after 8 weeks of dietary scGOS/lcFOS supplementation during arthritis. In addition, we found a beneficial effect of the scGOS/lcFOS diet on BMD in arthritic mice. However, despite these positive effects on bone and the microbiota composition, the scGOS/lcFOS diet did not induce a change in Th cell subsets or a reproducible therapeutic effect on the progression of autoimmune arthritis in IL-1Ra^-/-^ mice. Altogether, despite the lack of anti-rheumatic effects, this study suggests the ability of scGOS/lcFOS supplement to alter the gut microbiota into a more beneficial state and improving the bone mineral density.

## Supporting information

S1 Fig Dietary supplementation with scGOS/lcFOS has no effect on bacterial richness and diversity. (A) Chao index1, (B) Shannon index, (C) PD whole tree are shown. (D) Principal coordinates analysis (PCoA) based on an unweighted UniFrac analysis of the intestinal microbial composition. The position and distance of data points indicates the degree of similarity in terms of both presence and relative abundance of bacterial taxonomies. Data (mean + SEM) represent 16S rRNA gene 454-pyrosequencing analysis of intestinal microbiota of of IL-1Ra-/- mice fed a control diet (n = 8) or a diet containing either 1% (n = 7) or 2.5% (n = 8) scGOS/lcFOS for 8 weeks.
(TIF)

S2 Fig Diet containing scGOS/lcFOS has no effect on Thelper cell subsets in joint draining lymph nodes. Dot plots showing percentage of IFNγ+ Th1 (A) IL-4+ Th2 (B) IL-17+ Th17 (C) and FoxP3+ Treg cells among CD3+CD4+ cells isolated from the joint draining lymph nodes of arthritic IL-1Ra-/- mice. The mice were on either 2.5% or 5% scGOS/lcFOS diet or were fed a control diet. No significant differences as tested by Kruskal-Wallis with Dunn’s post test.
(TIF)

S3 Fig Prebiotic scGOS/lcFOS diet improves the overall bone mineral density in arthritic IL-1Ra deficient mice. (A) Bone mineral density (BMD) and (B) Bone mineral content (BMC) of arthritic IL-1Ra^-/-^ mice. Dual-energy X-ray absorptiometry (DEXA) scanning was performed after 10 weeks of dietary treatment with either 2.5% or 5% scGOS/lcFOS. *p<0.05 by Kurskal-Wallis with Dunn’s post test.
(TIF)

S4 Fig scGOS/lcFOS diet has no effect on Th cells subsets in IL-1Ra-/- mice. Gene expression of FoxP3, RORγt and Tbet in ileum (A), mesenteric lymph nodes (B), spleen (C) and colon (D) of IL-1Ra-/- mice fed a diet containing either 1%, 2.5% or 5% scGOS/lcFOS. Relative mRNA expression is shown as 2-ΔCt *10000, corrected for GAPDH. No significant differences as tested by Kruskal-Wallis with Dunn’s post test.
(TIF)

S5 Fig Intestinal T helper cells subsets are not affected by scGOS/lcFOS containing diet. Dot plots showing percentage of IFNγ+ Th1 (A) IL-4+ Th2 (B) IL-17+ Th17 (C) and FoxP3+ Treg (D) cells among CD3+CD4+ cells isolated from the small intestine lamina propria of arthritic IL-1Ra-/- mice. The mice were on either 2.5% or 5% scGOS/lcFOS diet or were fed a control diet. No significant differences as tested by Kruskal-Wallis with Dunn’s post test.
(TIF)

S1 Table Relative abundance on family and genus level in IL-1Ra-/- mice fed either a control diet or a diet containing 1.0% or 2.5% short-chain galaco-oligosaccharides / fructo-oligosaccharides (scGOS/lcFOS). Significant alterations by Mann-Whitney U (MWU) after Benjamini-Hochberg correction (FDR) for multiple testing are in bold. The color blue indicates an increase in the treatment group compared to the control group, while the color red indicates a decrease.
(DOCX)

S2 Table Prebiotic diet containing scGOS/lcFOS alteres the composition of intestinal microbiota IL-1Ra-/- mice. Relative abundance on family and genus level in IL-1Ra-/- mice fed either a control diet or a diet containing 1.0% or 2.5% short-chain galaco-oligosaccharides / fructo-oligosaccharides (scGOS/lcFOS). Significant alterations by Mann-Whitney U (MWU) after Benjamini-Hochberg correction (FDR) for multiple testing are in bold. The color blue indicates an increase in the treatment group compared to the control group, while the color red indicates a decrease.
(DOCX)

S1 File Supportive information file. Excel file containing the raw data underlying the results of this manuscript that lead to Figs 2 and 3 and S2–S5 Figs.
(XLSX)
