# Correction: Supplementation of diet with non-digestible oligosaccharides alters the intestinal microbiota, but not arthritis development, in IL-1 receptor antagonist deficient mice

**DOI:** 10.1371/journal.pone.0227517

**Published:** 2019-12-31

**Authors:** Rebecca Rogier, Thomas H. A. Ederveen, Harm Wopereis, Anita Hartog, Jos Boekhorst, Sacha A. F. T. van Hijum, Jan Knol, Johan Garssen, Birgitte Walgreen, Monique M. Helsen, Peter M. van der Kraan, Peter L. E. M. van Lent, Fons A. J. van de Loo, Shahla Abdollahi-Roodsaz, Marije I. Koenders

The following information is missing from the Funding statement: The Dutch Arthritis Society helped fund this study (grant 16-1-403).
