# Supplementary figures and images for: Supplementation of diet with non-digestible oligosaccharides alters the intestinal microbiota, but not arthritis development, in IL-1 receptor antagonist deficient mice
Source: PLoS One. 2019 Jul 8;14(7):e0219366. doi: 10.1371/journal.pone.0219366 (PMC6613703; doi:10.1371/journal.pone.0219366)

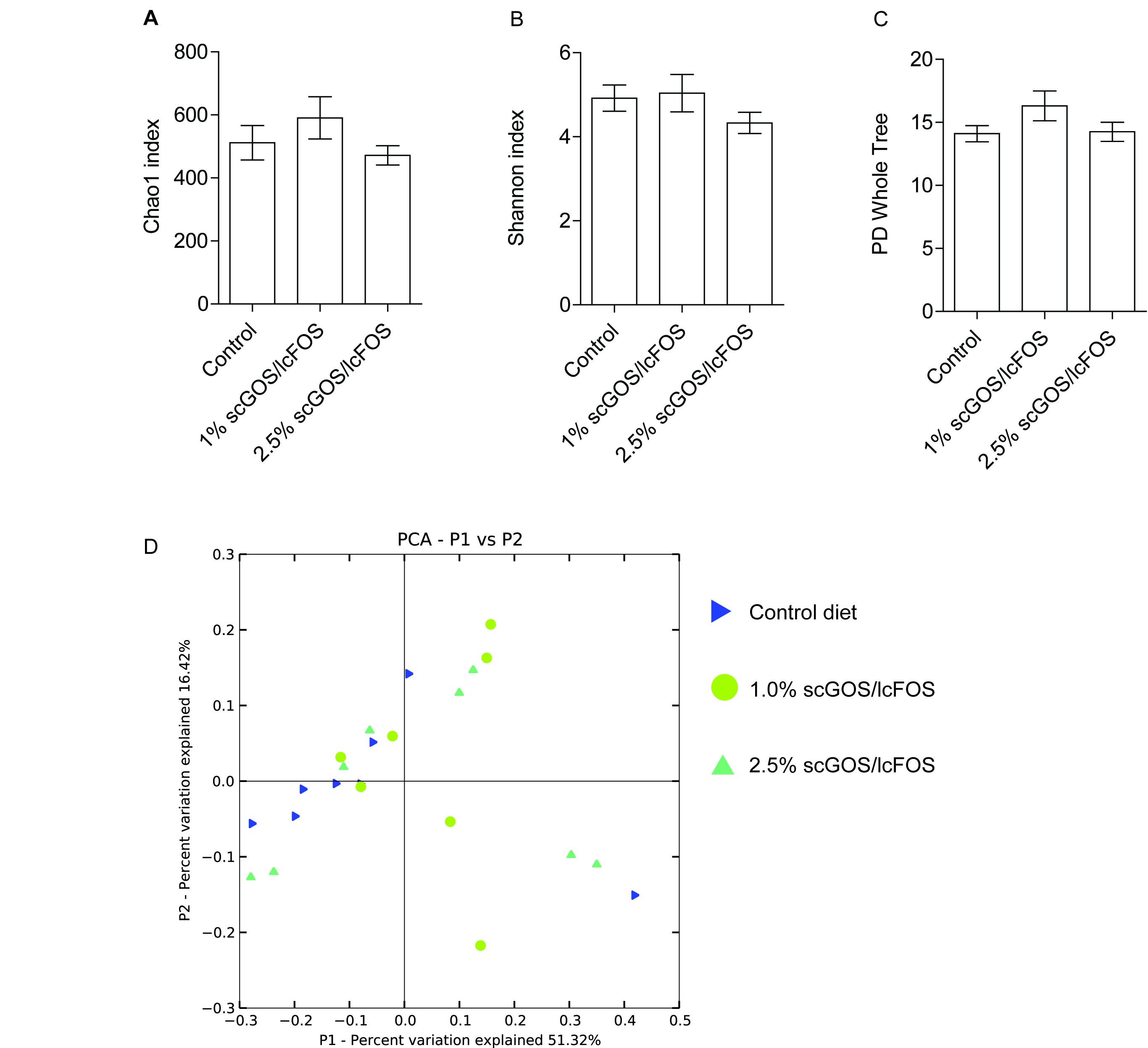

Supplement: S1 Fig — (A) Chao index1, (B) Shannon index, (C) PD whole tree are shown. (D) Principal coordinates analysis (PCoA) based on an unweighted UniFrac analysis of the intestinal microbial composition. The position and distance of data points indicates the degree of similarity in terms of both presence and relative abundance of bacterial taxonomies. Data (mean + SEM) represent 16S rRNA gene 454-pyrosequencing analysis of intestinal microbiota of of IL-1Ra-/- mice fed a control diet (n = 8) or a diet containing either 1% (n = 7) or 2.5% (n = 8) scGOS/lcFOS for 8 weeks. (TIF) [file pone.0219366.s001.tif]

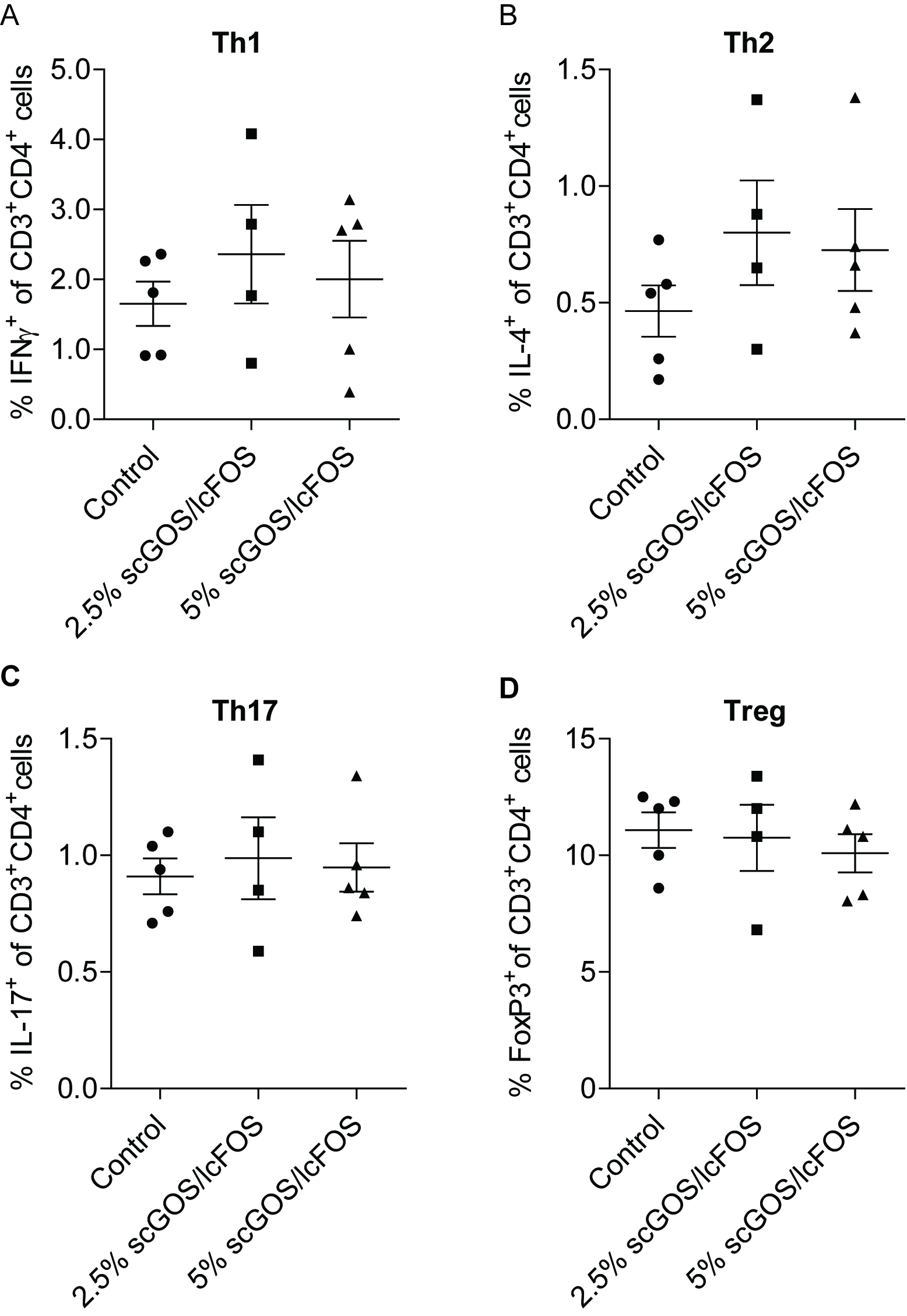

Supplement: S2 Fig — Dot plots showing percentage of IFNγ+ Th1 (A) IL-4+ Th2 (B) IL-17+ Th17 (C) and FoxP3+ Treg cells among CD3+CD4+ cells isolated from the joint draining lymph nodes of arthritic IL-1Ra-/- mice. The mice were on either 2.5% or 5% scGOS/lcFOS diet or were fed a control diet. No significant differences as tested by Kruskal-Wallis with Dunn’s post test. (TIF) [file pone.0219366.s002.tif]

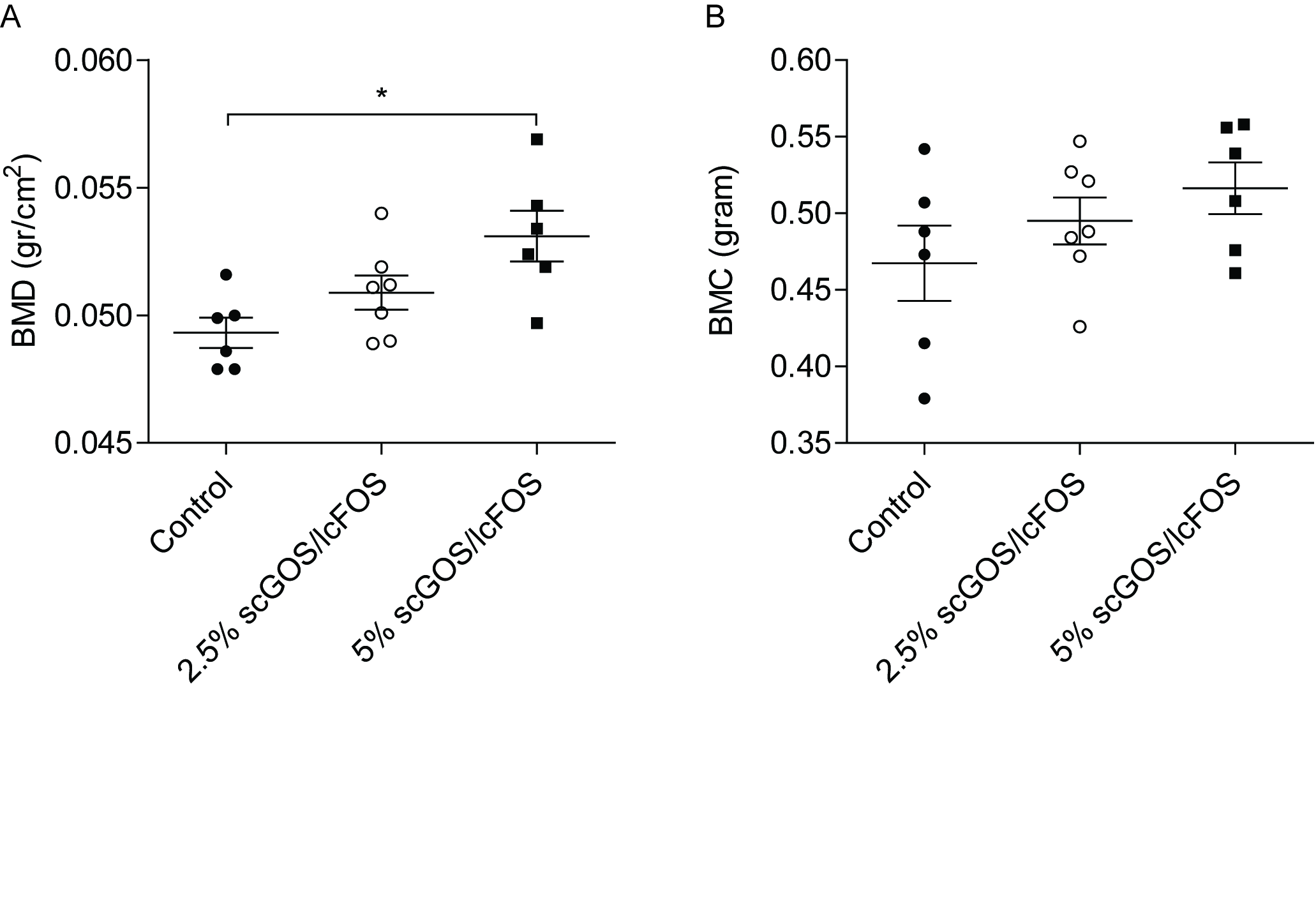

Supplement: S3 Fig — (A) Bone mineral density (BMD) and (B) Bone mineral content (BMC) of arthritic IL-1Ra-/- mice. Dual-energy X-ray absorptiometry (DEXA) scanning was performed after 10 weeks of dietary treatment with either 2.5% or 5% scGOS/lcFOS. *p<0.05 by Kurskal-Wallis with Dunn’s post test. (TIF) [file pone.0219366.s003.tif]

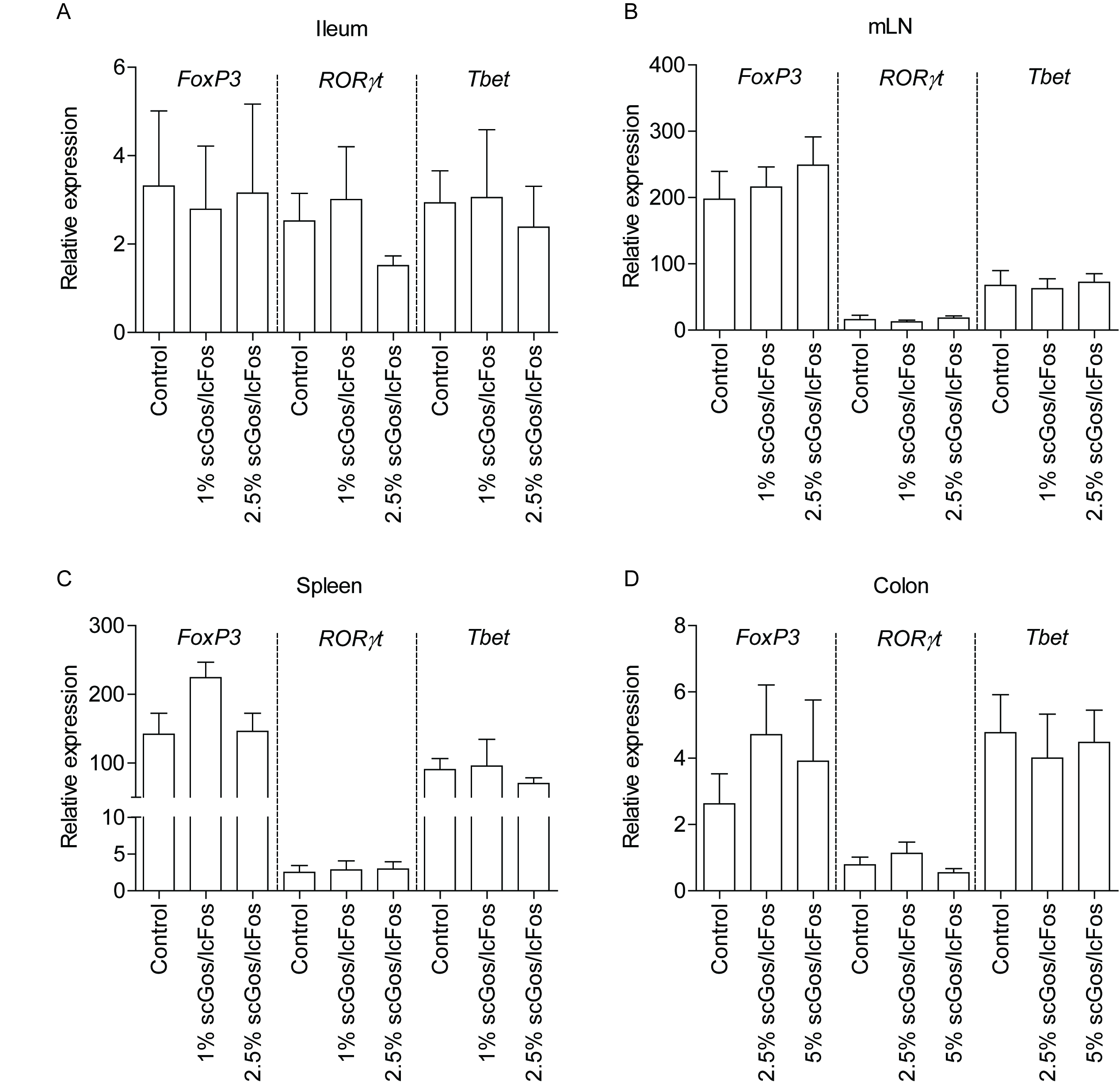

Supplement: S4 Fig — Gene expression of FoxP3, RORγt and Tbet in ileum (A), mesenteric lymph nodes (B), spleen (C) and colon (D) of IL-1Ra-/- mice fed a diet containing either 1%, 2.5% or 5% scGOS/lcFOS. Relative mRNA expression is shown as 2-ΔCt *10000, corrected for GAPDH. No significant differences as tested by Kruskal-Wallis with Dunn’s post test. (TIF) [file pone.0219366.s004.tif]

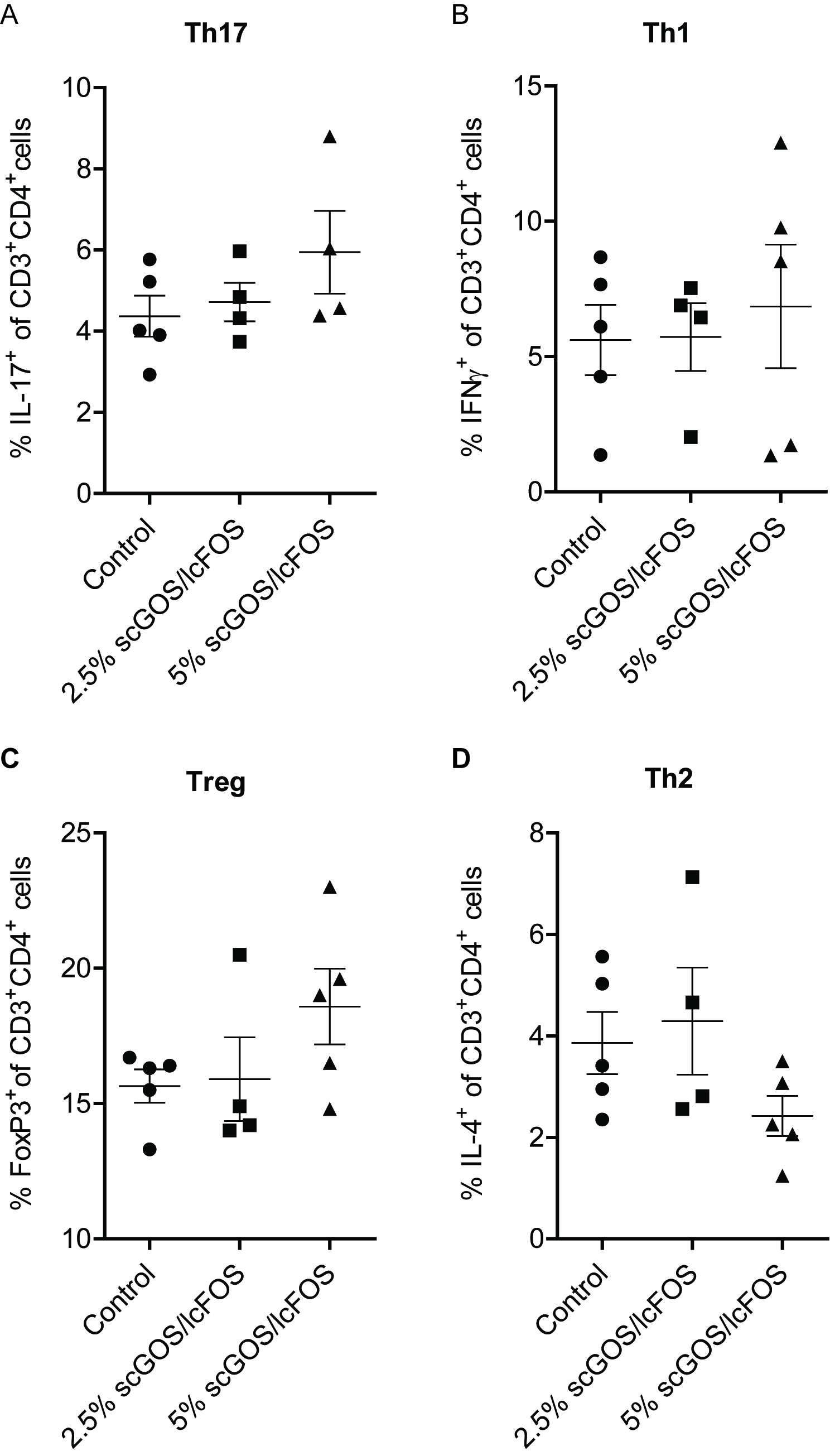

Supplement: S5 Fig — Dot plots showing percentage of IFNγ+ Th1 (A) IL-4+ Th2 (B) IL-17+ Th17 (C) and FoxP3+ Treg (D) cells among CD3+CD4+ cells isolated from the small intestine lamina propria of arthritic IL-1Ra-/- mice. The mice were on either 2.5% or 5% scGOS/lcFOS diet or were fed a control diet. No significant differences as tested by Kruskal-Wallis with Dunn’s post test. (TIF) [file pone.0219366.s005.tif]
