# Supplementary material for: Supplementation of diet with non-digestible oligosaccharides alters the intestinal microbiota, but not arthritis development, in IL-1 receptor antagonist deficient mice
Source: PLoS One. 2019 Jul 8;14(7):e0219366. doi: 10.1371/journal.pone.0219366 (PMC6613703; doi:10.1371/journal.pone.0219366)
Supplement: S1 Table — Significant alterations by Mann-Whitney U (MWU) after Benjamini-Hochberg correction (FDR) for multiple testing are in bold. The color blue indicates an increase in the treatment group compared to the control group, while the color red indicates a decrease. (DOCX) [file pone.0219366.s006.docx]

**S1 Table**. **The average and total number of (assigned) reads and operational taxonomic units (OTU) per experimental group.**

|  | Reads | | | OTU | | | Assigned at Phylum | | | Assigned at Genus | |  |
| --- | --- | --- | --- | --- | --- | --- | --- | --- | --- | --- | --- | --- |
| *Diet* | **Average** | **SEM** | **Total** | **Average** | **SEM** | **Total** | **Total Reads** | | **%** | **Total Reads** | **%** | **Group Size** |
| Control | 8555 | 578 | 68441 | 545 | 55 | 4362 | 67350 | 98.4% | | 33082 | 48.3% | n = 8 |
| 1.0% scGOS/lcFOS | 6544 | 644 | 45808 | 498 | 41 | 3487 | 44929 | 98.1% | | 22674 | 49.5% | n = 7 |
| 2.5% scGOS/lcFOS | 6796 | 298 | 54364 | 422 | 23 | 3379 | 53845 | 99.0% | | 22239 | 40.9% | n = 8 |

In addition, the number and percentage of reads assigned to phylum or genus level are shown.
