# Supplementary material for: Supplementation of diet with non-digestible oligosaccharides alters the intestinal microbiota, but not arthritis development, in IL-1 receptor antagonist deficient mice
Source: PLoS One. 2019 Jul 8;14(7):e0219366. doi: 10.1371/journal.pone.0219366 (PMC6613703; doi:10.1371/journal.pone.0219366)
Supplement: S2 Table — Relative abundance on family and genus level in IL-1Ra-/- mice fed either a control diet or a diet containing 1.0% or 2.5% short-chain galaco-oligosaccharides / fructo-oligosaccharides (scGOS/lcFOS). Significant alterations by Mann-Whitney U (MWU) after Benjamini-Hochberg correction (FDR) for multiple testing are in bold. The color blue indicates an increase in the treatment group compared to the control group, while the color red indicates a decrease. (DOCX) [file pone.0219366.s007.docx]

**S2 Table. Prebiotic diet containing scGOS/lcFOS alteres the composition of intestinal microbiota IL-1Ra^-/-^ mice.**

|  |  | **Relative abundance** | | | **Fold change** | |
| --- | --- | --- | --- | --- | --- | --- |
| **Taxon level** | **Taxon name** | **Control diet** | **1.0%** | **2.5%** | **Control diet vs. 1.0%** | **Control diet vs. 2.5%** |
| genus | Turicibacter | 8.37% | 0.02% | 0.01% | -8.92 | **-9.52** |
| family | Erysipelotrichaceae | 8.57% | 0.11% | 0.14% | -6.30 | **-5.93** |
| genus | Clostridium | 2.27% | 0.00% | 0.12% | na | **-4.23** |
| family | Clostridiaceae | 2.48% | 0.00% | 0.13% | na | **-4.23** |
| genus | Lactococcus | 0.10% | 0.05% | 0.01% | -1.15 | **-3.68** |
| family | Deferribacteraceae | 1.73% | 0.40% | 0.23% | -2.11 | -2.91 |
| genus | Mucispirillum | 1.73% | 0.40% | 0.23% | -2.11 | -2.91 |
| family | Streptococcaceae | 0.24% | 0.10% | 0.05% | -1.31 | **-2.39** |
| family | Incertae Sedis XIV | 0.11% | 9.00% | 0.02% | 6.37 | -2.25 |
| genus | Blautia | 0.11% | 9.00% | 0.02% | 6.37 | -2.25 |
| genus | Streptococcus | 0.13% | 0.05% | 0.04% | -1.43 | **-1.84** |
| genus | Lawsonia | 0.39% | 0.57% | 0.12% | 0.55 | -1.69 |
| genus | Oscillibacter | 1.39% | 0.77% | 0.44% | -0.86 | **-1.67** |
| family | Ruminococcaceae | 3.18% | 2.00% | 1.06% | -0.67 | **-1.59** |
| family | Coriobacteriaceae | 0.45% | 0.21% | 0.17% | -1.12 | **-1.44** |
| genus | Odoribacter | 0.25% | 0.37% | 0.12% | 0.56 | -1.12 |
| family | Desulfovibrionaceae | 0.71% | 0.95% | 0.33% | 0.41 | -1.09 |
| genus | Desulfovibrio | 0.31% | 0.35% | 0.21% | 0.17 | -0.59 |
| family | Helicobacteraceae | 3.13% | 1.13% | 2.08% | -1.47 | -0.59 |
| family | Rikenellaceae | 3.62% | 7.30% | 2.43% | 1.01 | **-0.57** |
| genus | Helicobacter | 3.05% | 1.12% | 2.07% | -1.44 | -0.56 |
| genus | Rikenella | 0.21% | 0.38% | 0.14% | 0.88 | -0.51 |
| family | TM7 sub sub sub | 0.02% | 0.15% | 0.02% | 2.65 | -0.45 |
| genus | TM7 genera incertae sedis | 0.02% | 0.15% | 0.02% | 2.65 | -0.45 |
| genus | Escherichia/Shigella | 0.03% | 0.10% | 0.02% | 1.64 | -0.39 |
| genus | Alistipes | 1.62% | 4.64% | 1.37% | 1.52 | -0.25 |
| family | Enterococcaceae | 9.05% | 4.34% | 7.63% | -1.06 | **-0.25** |
| genus | Enterococcus | 9.01% | 4.33% | 7.61% | -1.06 | **-0.24** |
| family | Enterobacteriaceae | 0.03% | 0.11% | 0.03% | 1.77 | -0.06 |
| family | Porphyromonadaceae | 3.99% | 8.77% | 5.34% | 1.14 | 0.42 |
| family | Lactobacillaceae | 5.32% | 7.14% | 8.15% | 0.42 | **0.62** |
| genus | Lactobacillus | 5.32% | 7.14% | 8.15% | 0.42 | **0.62** |
| family | Bacteroidaceae | 12.71% | 13.78% | 19.88% | 0.12 | 0.65 |
| genus | Bacteroides | 12.71% | 13.78% | 19.88% | 0.12 | 0.65 |
| genus | Parabacteroides | 0.59% | 0.97% | 1.01% | 0.71 | 0.77 |
| genus | Barnesiella | 0.24% | 0.29% | 0.65% | 0.31 | **1.46** |
| family | Lachnospiraceae | 6.50% | 14.57% | 27.97% | 1.17 | **2.11** |
| family | Alcaligenaceae | 0.22% | 0.04% | 0.00% | -2.41 | na |
| genus | Parasutterella | 0.21% | 0.04% | 0.00% | -2.54 | na |

Relative abundance on family and genus level in IL-1Ra^-/-^ mice fed either a control diet or a diet containing 1.0% or 2.5% short-chain galaco-oligosaccharides / fructo-oligosaccharides (scGOS/lcFOS). Significant alterations by Mann-Whitney U (MWU) after Benjamini-Hochberg correction (FDR) for multiple testing are in bold. The color blue indicates an increase in the treatment group compared to the control group, while the color red indicates a decrease.
